# Catalytic dye degradation by novel phytofabricated silver/zinc oxide composites

**DOI:** 10.3389/fchem.2022.1013077

**Published:** 2022-10-31

**Authors:** Khalida Bloch, Shahansha M. Mohammed, Srikanta Karmakar, Satyajit Shukla, Adersh Asok, Kaushik Banerjee, Reshma Patil-Sawant, Noor Haida Mohd Kaus, Sirikanjana Thongmee, Sougata Ghosh

**Affiliations:** ^1^ Department of Microbiology, School of Science, RK University, Rajkot, Gujarat, India; ^2^ Functional Materials Section (FMS), Materials Science and Technology Division (MSTD), CSIR-National Institute for Interdisciplinary Science and Technology (NIIST), Council of Scientific and Industrial Research (CSIR), Thiruvananthapuram, Kerala, India; ^3^ Department of Polymer Science and Technology, Calcutta University, Kolkata, West Bengal, India; ^4^ Academy of Scientific and Innovative Research (AcSIR), Ghaziabad, India; ^5^ Photosciences and Photonics Section, Chemical Sciences and Technology Division (CSTD), CSIR-National Institute for Interdisciplinary Science and Technology (NIIST), Council of Scientific and Industrial Research (CSIR), Thiruvananthapuram, Kerala, India; ^6^ National Referral Laboratory, ICAR-National Research Centre for Grapes, Pune, India; ^7^ School of Chemical Sciences, Universiti Sains Malaysia, Penang, Malaysia; ^8^ Department of Physics, Faculty of Science, Kasetsart University, Bangkok, Thailand

**Keywords:** *Plumbago auriculata*, zinc oxide particles, silver mixed composites, phytochemical, methylene blue, photocatalysis

## Abstract

Phytofabrication of the nanoparticles with exotic shape and size is an attractive area where nanostructures with noteworthy physicochemical and optoelectronic properties that can be significantly employed for photocatalytic dye degradation. In this study a medicinal plant, *Plumbago auriculata* leaf extract (PALE) was used to synthesize zinc oxide particles (ZnOPs) and silver mixed zinc oxide particles (ZnOAg1Ps, ZnOAg10Ps, ZnO10Ag1Ps) by varying the concentration of the metal precursor salts, i.e. zinc acetate and silver nitrate. The PALE showed significantly high concentrations of polyphenols, flavonoids, reducing sugar, starch, citric acid and plumbagin up to 314.3 ± 0.33, 960.0 ± 2.88, 121.3 ± 4.60, 150.3 ± 3.17, 109.4 ± 2.36, and 260.4 ± 8.90 μg/ml, respectively which might play an important role for green synthesis and capping of the phytogenic nanoparticles. The resulting particles were polydispersed which were mostly irregular, spherical, hexagonal and rod like in shape. The pristine ZnOPs exhibited a UV absorption band at 352 nm which shifted around 370 in the Ag mixed ZnOPs with concomitant appearance of peaks at 560 and 635 nm in ZnO10Ag1Ps and ZnOAg1Ps, respectively. The majority of the ZnOPs, ZnOAg1Ps, ZnOAg10Ps, and ZnO10Ag1Ps were 407, 98, 231, and 90 nm in size, respectively. Energy dispersive spectra confirmed the elemental composition of the particles while Fourier transform infrared spectra showed the involvement of the peptide and methyl functional groups in the synthesis and capping of the particles. The composites exhibited superior photocatalytic degradation of methylene blue dye, maximum being 95.7% by the ZnOAg10Ps with a rate constant of 0.0463 s^−1^ following a first order kinetic model. The present result clearly highlights that Ag mixed ZnOPs synthesized using *Plumbago auriculata* leaf extract (PALE) can play a critical role in removal of hazardous dyes from effluents of textile and dye industries. Further expanding the application of these phytofabricated composites will promote a significant complementary and alternative strategy for treating refractory pollutants from wastewater.

## 1 Introduction

Recent industrial revolution has led to environmental pollution that has created a great concern. Several industries associated with dye, textiles, tanning of leather, paper and pulp are common sources of refractory pollutants in the water bodies. Discharge of the dye contaminated effluents cause soil, ground and surface water pollution (Mani and Bharagava, 2017; Din et al., 2021). Since 4000 years, textile dying technology exists although in ancient times, natural dyes were predominantly used. In 1856, first synthetic dye named “mauveine” was synthesized by William E. Perkin employing oxidation of impure aniline (Fagier, 2021). After the discovery of diazotization and azo coupling, majority of synthetic dyes were developed. Around thousand dyes are commercially available today. Approximately 10,000 different dyes and pigments are used which requires production of 0.7 million tons of synthetically generated dyes every year across the globe (Hashmi et al., 2021). These hazardous dyes cause majority of water pollution and inhibit the photosynthetic activity of aquatic plants due to reduction in the penetration of sunlight. It induces the toxic effect on aquatic life as dye contains aromatic, heavy metals and chloride (Saraf et al., 2015). The dye contaminated water also causes soil pollution and adversely affects the germination of seed (Kumar et al., 2015). The synthetic dyes show harmful effects on animals and humans. Evaporation of chemicals from the dye effluents into air leads to allergic reaction as they are absorbed by skin (Saleh and Djaja, 2014).

Several chemical and physical methods such as adsorption, precipitation, flocculation, electrolysis, oxidation, reduction, electrochemical treatment, ion exchange are used for the removal of dyes from polluted water (Gautam et al., 2017). But these methods are complicated, expensive and are not ecofriendly. Hence, removal of dyes by biological methods is considered as promising alternative due to their cost effective, less sludge producing and ecofriendly nature (Bloch et al., 2020). Several microbes such as bacteria, fungi and algae are also used to degrade wide range of dyes (Daneshvar et al., 2007). Nanotechnology driven solutions for dye removal are more efficient owing to the smaller size, significantly large ratio for surface area to volume and exotic physico-chemical and opto-electronic properties of nanoparticles (Mandeep and Shukla, 2020). Synthesis of nanomaterials includes various physical, chemical and biological methods (Nitnavare et al., 2022). Nanoparticles synthesized using chemical and physical methods have several disadvantages as compared to biological synthesis since, they employ toxic and corrosive chemicals for reduction and stabilization (Ghosh et al., 2016). Microorganisms mediated syntheses of nanoparticles are slower and give only a limited number of morphological feature (Ghosh et al., 2022).

The use of medicinal plants for nanoparticles synthesis has acquired a great attention due to the ease in scaling up for larger production, apart from being cost effective and environmental friendly (Jamdade et al., 2019). Several nanoparticles composed of elemental gold, silver, copper, platinum, and palladium are synthesized using plants which show antimicrobial, antifungal, antibiofilm, cytotoxic, and photocatalytic properties (Shende et al., 2018). Novel nanocomposites of silver-loaded mesoporous zinc oxide and silver-titania are recently reported from extracts of *Lycium barbarum* L. for photocatalytic and therapeutic applications (Sharwani et al., 2021; Sharwani et al., 2022). Likewise, biogenic fabrication of silver nanoparticle-modified zeolitic imidazolate framework was reported as ideal for designing a non-enzymatic electrochemical sensor (Mohammad et al., 2022). Several carbon-based metal nanocomposites can also be used for photocatalytic degradation of hazardous dyes (Khan, 2021). In other studies biogenic zinc oxide nanoparticles (ZnONPs) synthesized from bulb and leaf extract of *Costus woodsonii* exhibited narrow band gap (Khan et al., 2019a,b).

On the basis of ethno botanical knowledge, *Plumbago auriculata* is widely used medicinal plant that possesses many secondary metabolites in its root and aerial parts (Govindan et al., 2020). Phytochemicals such as phenols, flavonoids, alkaloids, carbohydrates and saponins present in *P. auriculata* may help in synthesis of nanoparticles and may act as stabilizing and capping agent. The leaf and stem are rich in plumbagin, a napthoquinone that possesses antimicrobial, antibiofilm, anticancer and antifungal activities (Singh et al., 2018). The present study attempted to synthesize zinc oxide particles (ZnOPs) and silver mixed zinc oxide particles (ZnOAgPs) using *P. auriculata* leaf extract (PALE) for photocatalytic dye degradation.

## 2 Materials and methods

### 2.1 Collection of plant material and plant extract preparation

The plant specimen of *P. auriculata* along with flower was collected from the campus of RK. University, Rajkot, India. The plant was authenticated by taxonomist of Department of Bioscience, Saurashtra University, Rajkot, India. The collected *P. auriculata* leaves were washed thoroughly and were shade dried at ambient temperature for 10 days. Dehydrated leaves were pulverised to fine powder using electrical blender. A 5 g of dry leaf powder was added in deionized water (100 ml) and heated at 60°C for 20 min to prepare PALE. A Whatman no 1 filter paper was used for filtering the extract. The recovered filtrate was stored at 4°C and the same batch was used during all further experiments.

### 2.2 Phytochemical analysis

#### 2.2.1 Total phenolic content

Folin-Ciocalteau reagent (0.5 ml) was added to PALE (3 ml) and incubated for 3 min at 25°C. Thereafter 2 ml of 7% sodium carbonate (Na_2_CO_3_) was added followed by incubation at 100°C for 1 min in a boiling water bath. The absorbance was recorded at 650 nm (Luximon-Ramma et al., 2002). A standard gallic acid curve with concentration ranging from 10 to 100 μg/ml was used to determine the total phenolic content of PALE.

#### 2.2.2 Total flavonoid content

Initially 2% aluminium chloride (2 ml) dissolved in methanol was mixed with equal volume of PALE. The reaction mixture was incubated in darkness at 25°C for 10 min. The absorbance measured at 368 nm was extrapolated on a standard quercetin curve prepared in a range from 10 to 100 μg/ml for estimating the total flavonoid content (Wolfe et al., 2003).

#### 2.2.3 Total reducing sugar content

The 3, 5-Dinitrosalicylic acid (DNSA) reagent was mixed with equal volume (1 ml) of PALE and incubated for 5 min at 100°C. Thereafter, 10 ml of deionized water was added followed by recording of the absorbance at 540 nm (Miller et al., 1959). A standard glucose curve prepared in a range from 100 to 1000 μg/ml was used to find the total reducing sugar in PALE.

#### 2.2.4 Total starch content

Total starch content was estimated using anthrone reagent. PALE (1 ml) was added in anthrone reagent (4 ml) followed by incubation at 100°C for 8 min. After cooling the absorbance was recorded at 630 nm. The standard glucose curve (10–100 μg/ml) was used for the estimation of total starch content (Thayumanavan et al., 1984).

#### 2.2.5 Total citric acid content

The citric acid was estimated as per protocol reported by Saffaran et al. (1948). In brief, 5% trichloroacetic acid and PALE were mixed in equal volume (0.5 ml) to which 4 ml of acetic anhydride was added followed by incubation at 60°C for 10 min. In the next step pyridine (0.5 ml) was mixed and incubated at 60°C for 40 min. Reaction mixture was further allowed to incubate in ice bath. After 5 min total citric acid content in PALE was evaluated by recording the absorbance 540 nm and extrapolating on a citric acid standard curve prepared in a range from 10 to 400 μg/ml.

#### 2.2.6 Total ascorbic acid content

The PALE (4 ml) was initially brominated using 250 μL of bromine water followed by 250 μL of thiourea. Further 1 ml of 2, 4− Dinitrophenylhydrazine (DNPH) was added and was incubated at room temperature for 3 h. Then, 85% sulphuric acid (7 ml) was added and the absorbance was recorded at 540 nm. Standard ascorbic acid (1–10 μg/ml) was used to determine total ascorbic acid content (Sadasivam et al., 2008).

#### 2.2.7 Total plumbagin content

Total plumbagin content was estimated by adding 10% alcoholic potassium hydroxide (KOH) into equal volume (0.5 ml) of PALE in which 1.5 ml of absolute alcohol was further added. Total plumbagin content was estimated by recording the absorbance at 520 nm and using a standard curve prepared by varying plumbagin concentration from 100 to 1000 μg/ml (Israni et al., 2010).

### 2.3 Synthesis of zinc oxide particles

At first, a stock solution of 10 mM of zinc acetate (ZnC_4_H_6_O_4_) salt was prepared by mixing it in 100 ml of deionized water under stirring condition. Then, 5 ml of PALE was added to the 95 ml of ZnC_4_H_6_O_4_ stock solution and stirred again at 150 rpm for 24 h at room temperature. After incubation, brown coloured pellets were collected by centrifuging the reaction mixture for 20 min at 10,000 rpm. The pellets were washed twice with distilled water by alternate centrifugation and redispersion for three times. The collected pellets were allowed to calcinate in muffle furnace at 400°C for 4 h (Naseer et al., 2020).

### 2.4 Synthesis of Ag mixed zinc oxide particles

Silver (Ag) mixed ZnOPs were fabricated by varying the concentration of AgNO_3_ and ZnC_4_H_6_O_4_. The salt solutions were mixed in identical concentration of 1 and 10 mM for the synthesis of ZnOAg1Ps and ZnOAg10Ps, respectively. For the synthesis of ZnO10Ag1Ps, 10 mM of ZnC_4_H_6_O_4_ was mixed with 1 mM of AgNO_3_. Synthesis of respective particles was carried out by mixing 5 ml PALE into 95 ml of each reaction mixture under stirring condition (150 rpm for 24 h). The synthesized particles were recovered and washed similar to the process discussed above. The collected pellets were further calcinated at 400°C for 4 h in a muffle furnace (Thakur et al., 2020).

### 2.5 Characterization

At first, the UV-Vis. Absorption spectra of the bio-synthesized particles were analyzed by using UV-Visible spectrometer (UV-1900, Shimadzu, Japan) operating at a resolution of 1 nm. The photoluminescence properties of the synthesized samples were recorded with Fluorescence Spectrophotometer (F-4700, Hitachi, Japan) at an excitation wavelength (*λ*_*ex*_) of 340 nm (Jangir et al., 2017). The surface morphology of particles was determined using transmission electron microscopy (TEM, Tecnai G2 F30, Thermo Fisher, United States) operating at an accelerating voltage of 300 kV. Before that a droplet of the prepared sonicated sample was placed directly on the carbon-coated copper grid that was subsequently dried at 37°C (Basri et al., 2020). Further, energy dispersive X-ray analysis (EDAX) was employed for determining the elemental composition using Octane ELITE T70, EDAX, United States equipped in the TEM. Hydrodynamic size of the synthesized particles was estimated employing dynamic light scattering in a Particle Size/Zeta Potential Analyzer, Microtrac, United States (Salunke et al., 2014). The crystalline phases present were determined using the X-ray diffraction (XRD, EMPYREAN 3, Malvern Panalytical B.V. Netherlands). The broad-scan analysis was typically conducted within the range of 10–90^0^ using the Cu *Kα* (λ_Cu_ = 1.540 Å) X-radiation. The functional groups associated with the particles were characterized by Fourier-transform infrared (FT-IR) using ThermoNIColet FTIR spectrophotometer (Nicolet Instrument, Thermo scientific, United States). The washed particles sample (20 µL) was mixed with 20 mg KBr and was dried at 50°C in hot air oven. The mixed powder was exposed to infrared source of 500–4500 cm^−1^ (Brus, 1986).

### 2.6 GC-MS/MS analysis

The analysis was performed using an Agilent GC (7890A) equipped with a CTC Combipal (CTC Analytics, Switzerland) autosampler, connected to a triple quadrupole mass spectrometer (7000B, Agilent Technologies, Santa Clara, United States). The instrument was controlled by Mass Hunter software (ver. B.05.00.412). The multi-mode inlet (MMI) was operated in split mode. Later, 2 µL of the PALE sample was injected into a gooseneck liner (78.5 × 6.5 mm, 4 mm). Two HP-5MS (5% phenylmethylpolysiloxane, Agilent Technologies, United States) capillary columns (15 m × 0.25 mm, 0.25 µm) were connected through a purged ultimate union (Backflush-PUU). Ultra-pure grade helium (BOC India Ltd., Kolkata) was used as the carrier gas, with a constant 1.2 ml/min flow. The oven temperature was programmed to start at 90°C (1 min hold), then ramped at 40°C/min to 170°C (0 min hold), followed by 15°C/min up to 290°C with a 6-min hold. This resulted in a total run time of 21 min. The temperatures of the ion source and transfer line were set to 230°C and 290°C respectively. Considering the number of molecules, it was important to optimise the chromatographic separation and mass spectrometric parameters to ensure the selectivity and sensitivity in analysis. The mass range was scanned from 50 to 550 amu. The identification of components was based on the comparison of their mass spectra with those of NIST mass spectral library.

### 2.7 Photocatalytic methylene blue dye degradation

The photocatalytic activity of ZnOPs and the ZnOAgPs was evaluated as the protocol reported earlier (Zachariah et al., 2008). In short, initially 0.4 g L^−1^ of each particle sample was added to 125 ml of 7.5 μM MB dye dissolved in distilled water with an initial neutral pH of 6.67. Thereafter, the resulting mixture was stirred to equilibrate stirring in the darkness (that is, without the UV irradiation) for 1 h in order to stabilize the adsorption of MB on the sample surface. The aqueous suspension was then subjected to UV irradiation in the photoreactor chamber (LZC-4X, Luzchem, Canada) using the 14 UVA lamps (6 top and 8 side lamps) with an emission peak intensity of 350 nm with concomitant magnetic stirring for 1 h. After each 10 min intervals, 8 ml aliquot was recovered and centrifuged (Hettich EBA 20, Sigma-Aldrich labware, Bengaluru, India). The supernatant was collected and examined using a UV-visible absorption spectrophotometer (UV-2401 PC, Shimadzu, Japan) to determine the residual MB concentration in the aqueous dye solution.

$$MB_{adsorbed}\left(\%\right)=\frac{C_{-60}-C_{0}}{C_{-60}}\times 100=\frac{A_{-60}-A_{0}}{A_{-60}}\times 100$$ (1)

where, *C*_*-60*_ and *C*_*0*_ are the MB concentrations within the aqueous solution before (time (*t*) = 60 min) and after (*t* = 0 min) the adsorption experiment conducted in the dark condition; while, *A*_*−60*_ and *A*_*0*_ are the corresponding absorbance values. The normalized concentration of MB remaining in the solution after stirring in the dark condition for 1 h is calculated using Eq. 2.

$$MB_{residual}\left(\%\right)=\frac{C_{0}}{C_{-60}}\times 100=\frac{A_{0}}{A_{-60}}\times 100$$ (2)

The normalized concentration of MB remaining in the solution under the UV irradiation is calculated using Eq. 3.

$$MB_{residual}\left(\%\right)=\frac{C_{t}}{C_{-60}}\times 100=\frac{A_{t}}{A_{-60}}\times 100$$ (3)

where, *C*_*t*_ is the MB concentration remaining within the aqueous dye solution after the UV irradiation time of *t* = *t* min; while, *A*_*t*_ is the corresponding absorbance value.

The first order kinetic constant (k) for the degradation of MB is calculated using Eq. 4

$$\ln\frac{C_{0}}{C_{t}}=kt$$ (4)

## 3 Results

### 3.1 Phytochemical analysis

The plants belonging to family of Plumbaginaceae are known to be a potential source of various phytochemicals such as phenols, flavonoids, napthoquinone, alkaloids, and carbohydrates that might play an important role in formation of particles by redox reaction. Table 1 shows high flavonoid content (960.0 ± 2.88 μg/ml) and phenolic content (314.3 ± 0.33 μg/ml) in PALE. The *Plumbago* is known to contain a napthoquinone called plumbagin which showed its presence even in PALE at a concentration of 260.0 μg/ml. Other phytochemicals such as citric acid, ascorbic acid and starch were also noted to be present in PALE. Hence, the presence of these phytochemicals may help in converting zinc acetate to ZnOPs.

**TABLE 1:** Phytochemicals content of PALE.

Phenolic (μg/ml)	Flavonoid (μg/ml)	Reducing sugar (μg/ml)	Starch (μg/ml)	Citric acid (μg/ml)	Ascorbic acid (μg/ml)	Plumbagin (μg/ml)
314.30 ± 0.33	960.0 ± 2.88	121.30 ± 4.60	150.30 ± 3.17	109.40 ± 2.36	1.97 ± 1.55	260.40 ± 8.90

### 3.2 UV-Visible absorption and photoluminescence spectroscopy analyses

**FIGURE 1:**
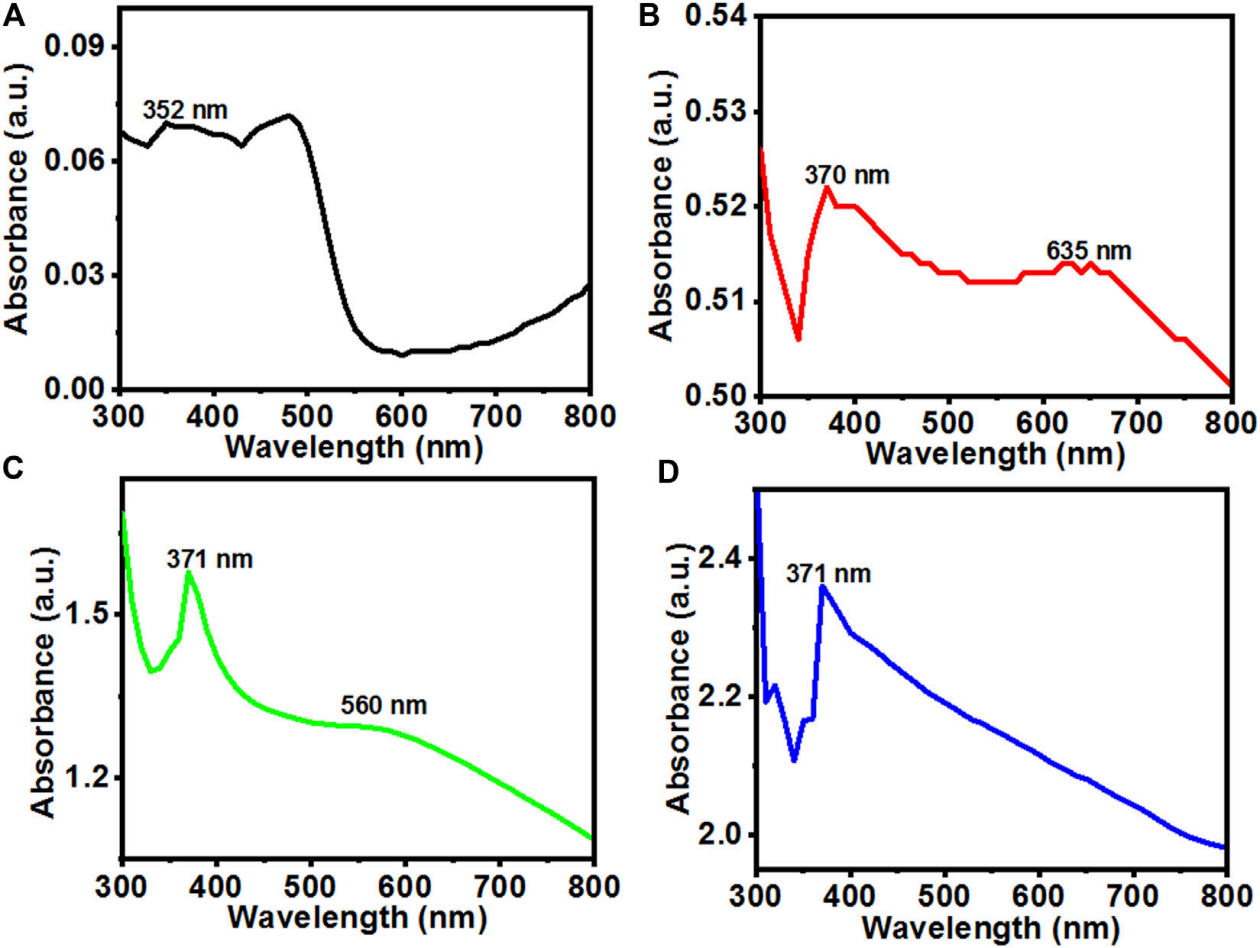
UV-Vis absorption spectra of the **(A)** ZnOPs; **(B)** ZnOAg1Ps; **(C)** ZnO10Ag1Ps; **(D)** ZnOAg10Ps synthesized by using PALE at room temperature.

Brown color precipitates were formed during reaction which converted to greyish white after calcination indicating synthesis of ZnOPs and ZnOAgPs. The UV-Vis absorption spectra of the synthesized particles were recorded as shown in Figures 1A–D. Figure 1A indicates the UV-Vis absorption spectrum of ZnOPs which showed a broad absorption in the range of 300–490 nm. The main characteristics peak of ZnOPs was observed at 352 nm. The slightly blue shifted peak position of ZnONPs with respect to bulk ZnO was observed implying lower particle size of ZnONPs in comparison to bulk ZnO.

On silver doping, the UV-Vis absorption spectrum of the ZnOAg1Ps revealed two characteristics peaks at 370 nm and 635 nm, respectively as revealed in Figure 1B. Further, with the increase of the concentration of salts in ZnO10Ag1Ps, the absorbance peak became prominent at ∼560 nm as evident in Figure 1C and blue shifted in comparison to that of ZnOAg1Ps. This blue shift may have arisen due to the decrease of optical scattering caused by grain growth and the reduction of grain boundary density (Lv et al., 2011). The UV-Vis absorbance spectrum of ZnOAg10Ps showed only the main characteristics peak of ZnOPs at 371 nm while there is no sharp peak observed for silver except some enhancement of visible absorbance as seen in Figure 1D. Here, the peak of ZnOPs is predominant over the peak of silver because during the preparation of the sample, the concentration of the zinc acetate was much higher as compared to that of silver nitrate. It is interesting to observe that the relative visible absorbance in the UV-Vis spectra of ZnOAg1Ps, ZnOAg10Ps, and ZnO10Ag1Ps increased significantly compared to that of pure ZnOPs. This increased visible absorbance indicates that these samples will be highly active in visible light thereby having promising photocatalytic dye degrading ability which is evaluated in the subsequent section.

**FIGURE 2:**
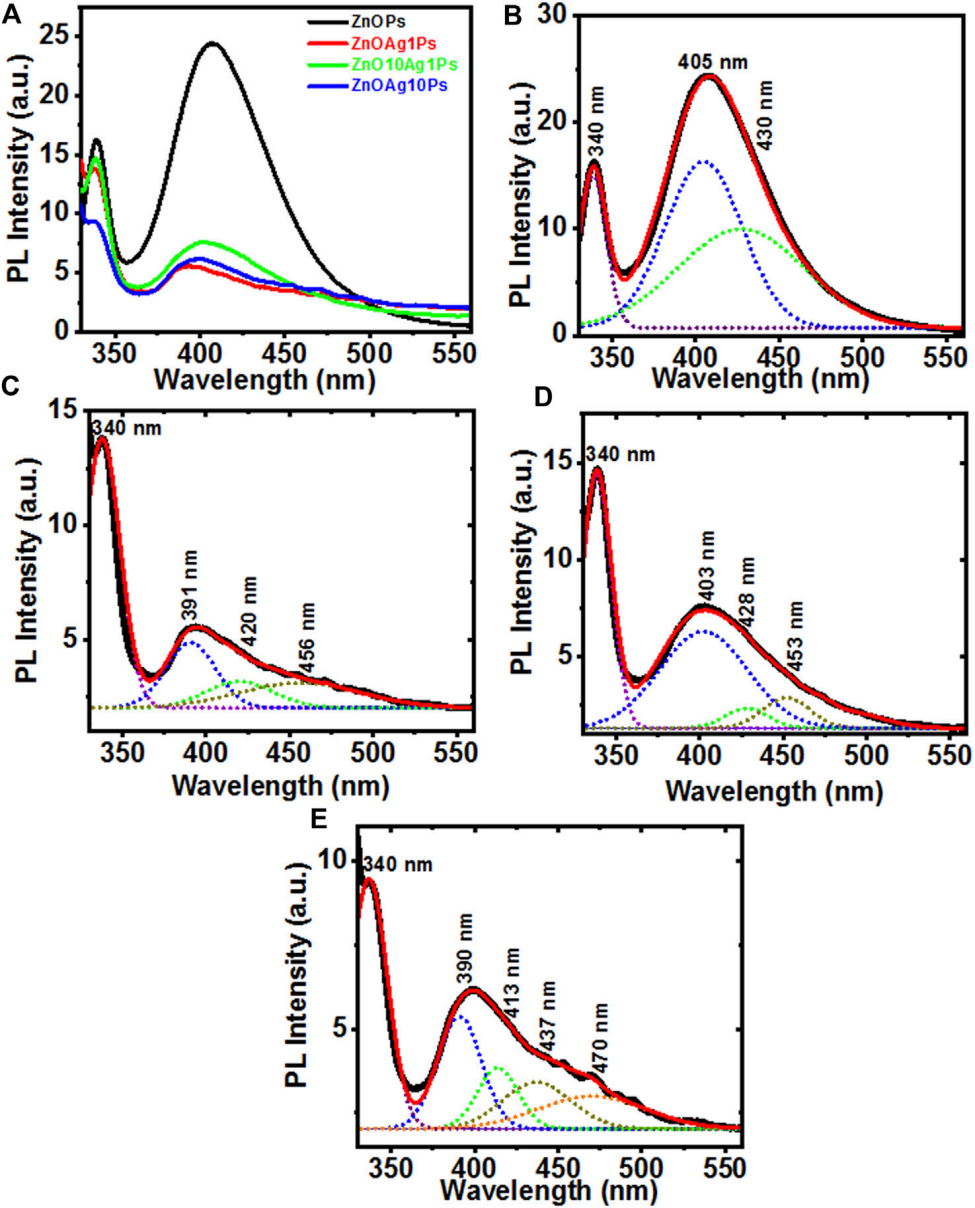
**(A)** Photoluminescence emission spectra of ZnOPs, ZnOAg1Ps, ZnOAg10Ps, ZnO10Ag1Ps; **(B–E)** the Gaussian fitted PL emission spectra of ZnOPs, ZnOAg1Ps, ZnOAg10Ps, ZnO10Ag1Ps, respectively.

Further, the movement, separation, and recombination of photo-generated electron and hole (e^−^−h^+^) pairs in the synthesized particles were measured by recording the room temperature PL emission spectra at 300 nm excitation which are shown in Figure 2A. The PL intensity of the ZnOAgPs decreased as compared to that of the ZnOPs which indicated an effective transfer of the interfacial charge from ZnO to Ag, serving as an electron sink that doesn’t allow the photo-induced carriers to recombine. Hence, the ZnOAgPs are estimated to show improved photocatalytic dye degradation performance than that of the pristine ZnOPs (Kadam et al., 2018). The Gaussian fitted PL emission spectra of the synthesized samples are shown in Figures 2B–E, respectively. It was observed that ZnOPs show a strong near band edge emission band that was centered at ∼340 nm in all samples. The Gaussian fitted deconvoluting PL emission spectra of the synthesized particles showed some additional bands at ∼405, ∼420, ∼456 and ∼470 nm wavelengths, that are specific to oxygen vacancy states (V_o_^+^ and V_o_^++^) which was singly and doubly charged as well as defects in ZnOPs (Saoud et al., 2015).

### 3.3 HRTEM image, DLS, and XRD analyses

**FIGURE 3:**
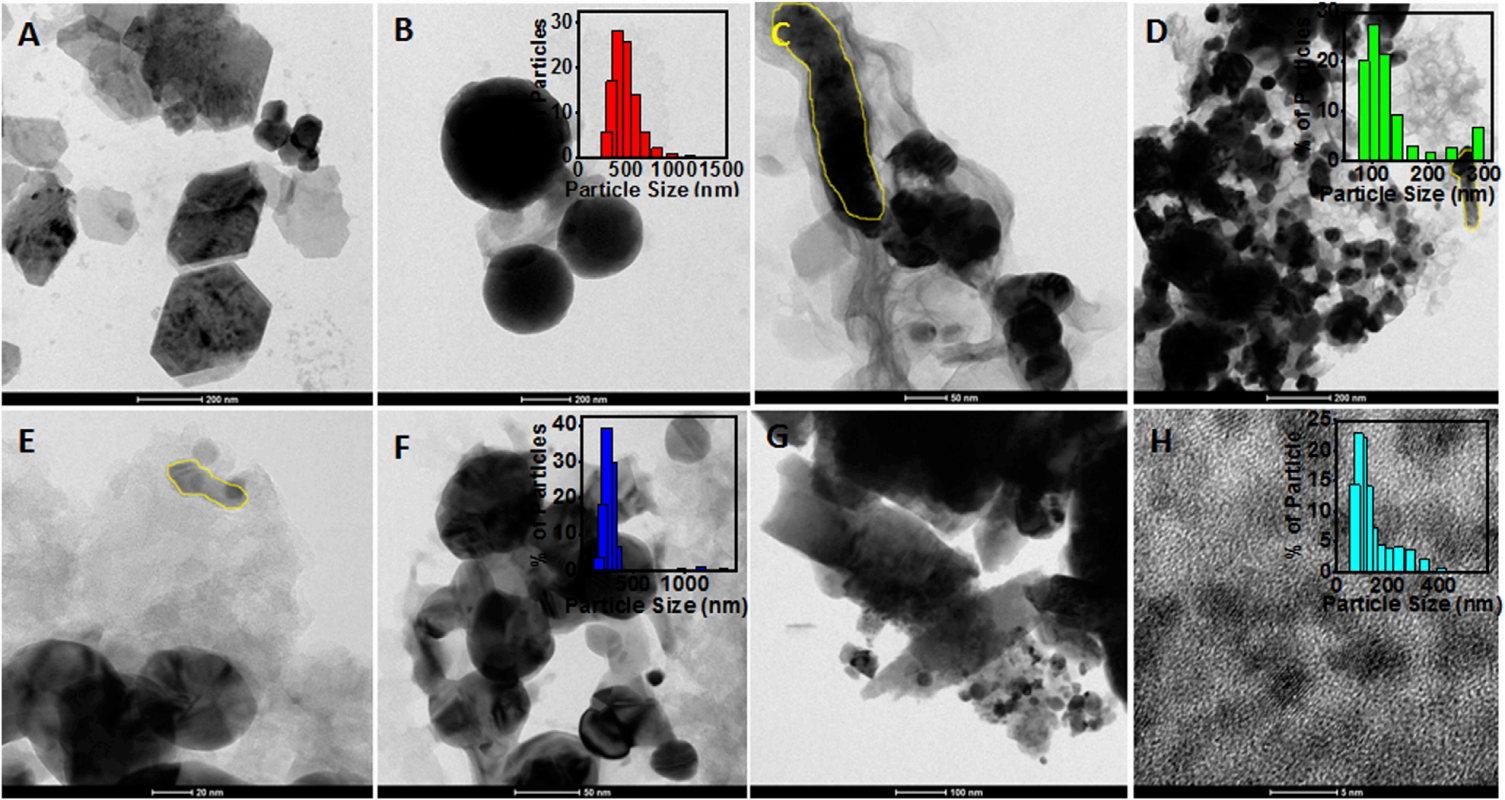
HRTEM images of particles synthesized using PALE. **(A)** ZnOPs with hexagonal shape (scale: 200 nm); **(B)** spherical shaped ZnOPs (scale: 200 nm) and inset showed the bar diagram of DLS data; **(C)** Rod shaped ZnOAg1Ps (scale: 50 nm); **(D)** Spherical ZnOAg1Ps (scale: 200 nm) and inset showed the bar diagram of DLS data; **(E)** Rod shaped ZnOAg10Ps (scale: 20 nm) and inset showed the bar diagram of DLS data; **(F)** spherical shaped ZnOAg10Ps (scale: 50 nm); **(G)** Rod shaped ZnO10Ag1Ps (scale: 100 nm) **(H)** spherical shaped ZnO10Ag1Ps (scale: 5 nm), and inset showed the bar diagram of DLS data.

The morphology of the synthesized particles was determined using HRTEM images, as shown in Figure 3. The HRTEM image of ZnOPs clearly showed two anisotropic microstructures of hexagonal and spherical (Figures 3A,B) shapes. The size and diameter of the observed hexagonal and spherical structures were observed to be 350 and 500 nm, respectively. It should be noted that the mentioned size of the particles are specific to the ones seen in the HRTEM image and not an average of several particles. The particle size of ZnOPs was also found by DLS analysis and the corresponding bar diagram is shown in the inset of Figure 3B. The size of ZnOPs was ranging from 289 to 1635 nm with maximum number of particles of size 407 nm. A nanorod of diameter ∼40 nm and length ∼350 nm was observed in ZnOAg1Ps sample (Figure 3C). There were some polydispersed spherical particles of size ∼50–200 nm as observed in ZnOAg1Ps also (Figure 3D). The particle size distribution of ZnOAg1Ps were in a range from 85 to 409 nm with majority of size ∼98 nm as seen in the inset of Figure 3D. Similar rods were also spotted for ZnOAg10Ps that were ∼50 × ∼10 nm in dimension as evident from Figure 3E. Apart from the rods, there were some spherical particles of size ∼30 nm as well. The size of the ZnOAg10Ps was ∼171–1635 nm with majority being around 231 nm as shown in inset of Figure 3F. A similar trend was also observed for ZnO10Ag1Ps where the rod shaped particles were larger in size (∼758 nm) as seen in Figures 3G,H. The size of the particles ranged from 72 to 687 nm most of which were around 90 nm.

**FIGURE 4:**
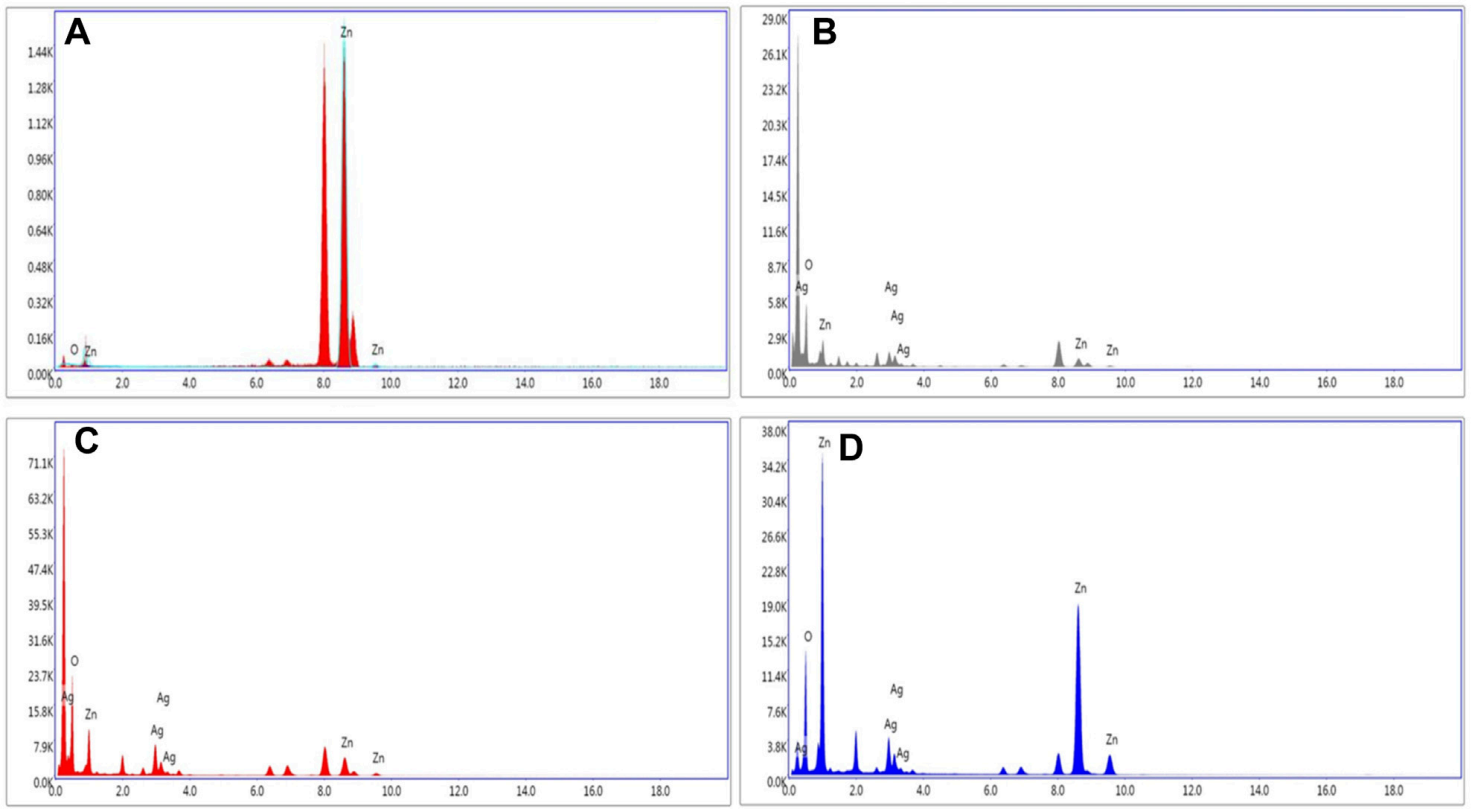
Representative spot EDS spectra of **(A)** ZnOPs; **(B)** ZnOAg1Ps; **(C)** ZnOAg10Ps; **(D)** ZnO10Ag1Ps.

The elemental zinc (Zn), oxygen (O), and silver (Ag) in the composites were confirmed by EDAX spectra as shown in Figure 4. The purity of ZnOPs was confirmed by the presence of Zn and O in Figure 4A. Figure 4B indicated the presence of Zn and Ag up to 16.8% and 16.6%, respectively in ZnOAg1Ps. Figure 4C showed 25.3% Zn and 24.6% Ag in ZnOAg10 whereas ZnO10Ag1Ps exhibited 57.2% Zn and 8.5% Ag as evident from Figure 4D.

**FIGURE 5:**
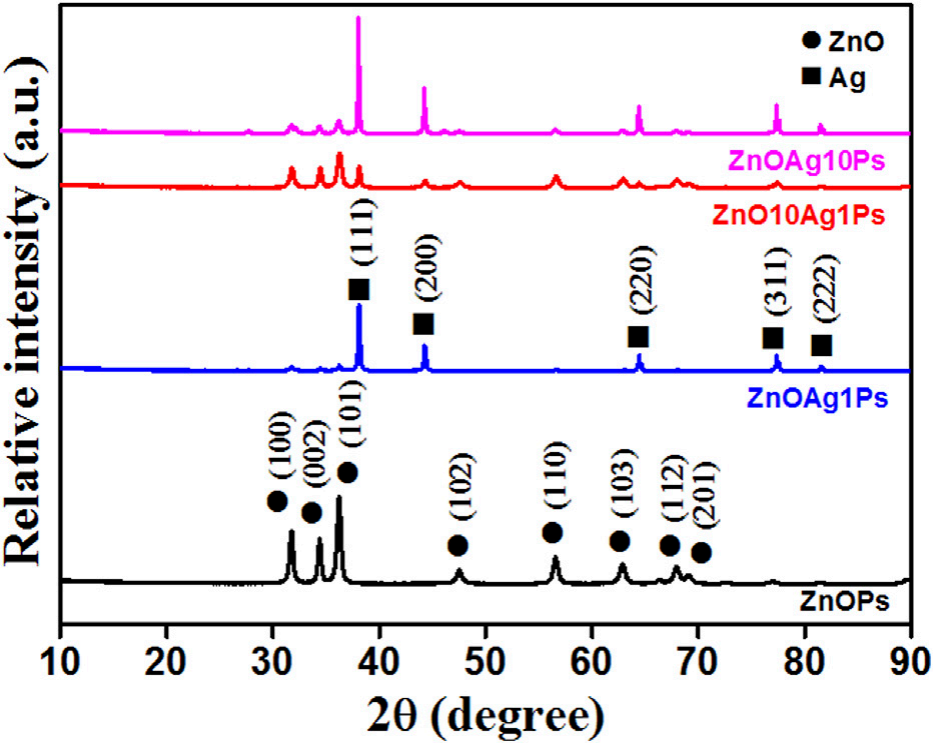
XRD patterns obtained for the different samples.

The XRD pattern obtained for the samples ZnOPs, ZnOAg1Ps, ZnO10Ag1Ps, and ZnOAg10Ps are presented in Figure 5. The obtained diffraction angle, the identified diffracting planes, the corresponding phases of ZnO (JCPDS file No.36-1451) and silver (JCPDS file No: 01-087-0597) are tabulated in Table 2. The planes of Ag are marked by * (star) for better clarification.

**TABLE 2:** Analysis of XRD patterns presented in Figure 5.

Sample	2θ (deg.)	Diffracting plane
ZnOPs	31.8	(100)
	34.4	(002)
	36.2	(101)
	47.5	(102)
	56.5	(110)
	62.8	(103)
	67.9	(112)
	69.1	(201)
ZnOAg1Ps	31.8	(100)
	34.5	(002)
	36.2	(101)
	38.1	*(111)
	44.3	*(200)
	56.5	(110)
	64.4	*(220)
	77.9	*(311)
	81.5	*(222)
ZnO10Ag1Ps	31.8	(100)
	34.4	(002)
	36.2	(101)
	38.1	*(111)
	44.3	*(200)
	47.5	(102)
	56.6	(110)
	62.8	(103)
	64.4	*(220)
	67.9	(112)
	69.1	(201)
	77.3	*(311)
	81.4	*(222)
ZnOAg10Ps	31.8	(100)
	34.4	(002)
	36.2	(101)
	38.1	*(111)
	44.3	*(200)
	56.6	(110)
	62.8	(103)
	64.4	(220)
	77.3	(311)
	81.4	(222)

Hence, the XRD pattern analyses of the synthesized samples confirmed the presence of ZnO and silver (Ag) nanoparticles in our synthesized samples.

### 3.4 FTIR spectroscopy analysis

**FIGURE 6:**
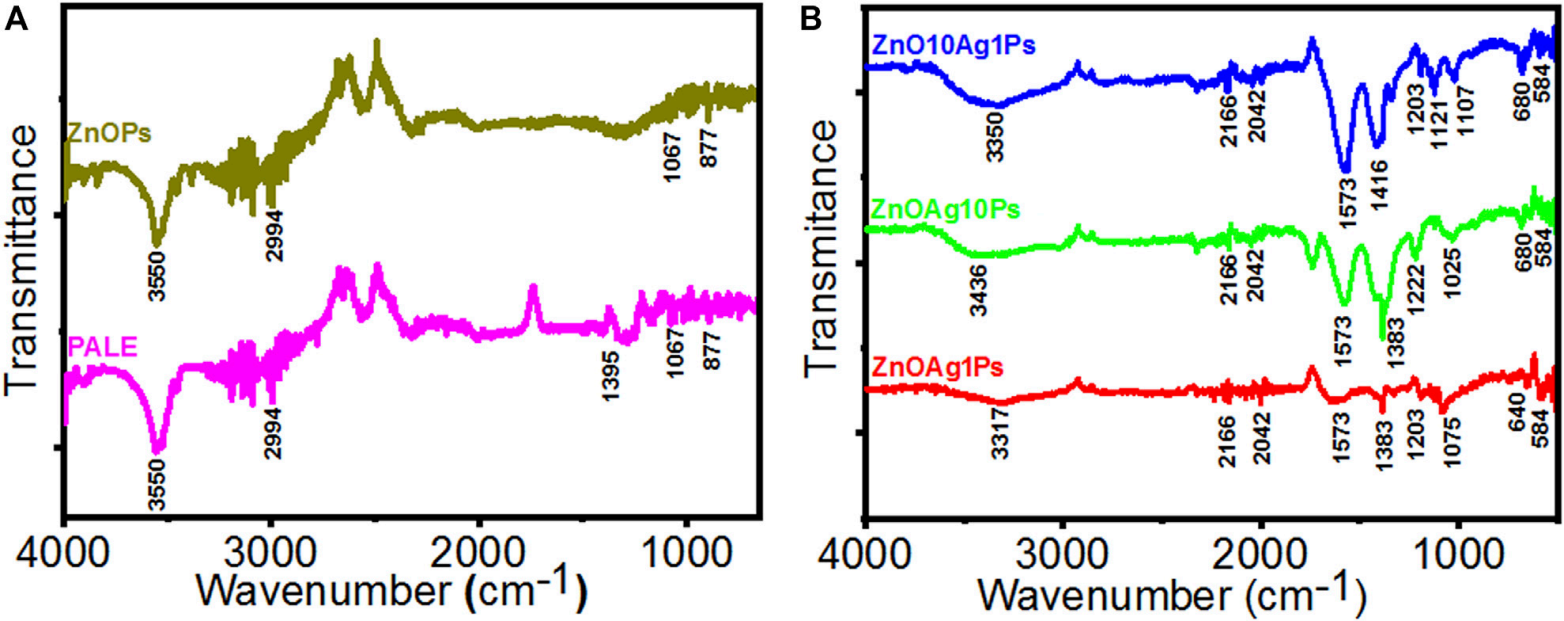
FTIR spectra of particles synthesized by PALE. **(A)** PALE and ZnOPs; **(B)** ZnOAg1Ps, ZnOAg10Ps, and ZnOAg10Ps.

The PALE comprises different phytochemicals such as phenolic, flavonoid, reducing sugar, starch, citric acid, ascorbic acid, plumbagin, *etc*, that played a significant role for reducing Ag^+^ and Zn^2+^ ions and stabilizing the synthesized particles. The PALE showed numerous characteristic bands in the FTIR spectra as depicted in Figures 6A,B. The peak at 3550 cm^−1^ is corresponding to the peptide linkage associated N–H stretch vibrations (Kannan and John, 2008), while the peak at 2994 cm^−1^ is specific to the stretching vibration of methyl groups (Li et al., 2007). The peak at 1395 cm^−1^ is attributed to germinal methyl group (Tripathy et al., 2010). The peak at 1067 cm^−1^ is attributed to the bending vibration of C–OH groups and the antisymmetric stretching band of polysaccharides and/or chlorophyll associated C–O–C groups (Jain et al., 2011). These peaks indicated the presence of different phytochemicals in the PALE extract. The FTIR spectra of the synthesized ZnOAgPs composites by PALE are shown in Figure 6B. The peak at 3550 cm^−1^, which is associated to the N–H stretch vibrations from the peptide linkages is slightly blue shifted for ZnAg1Ps and ZnAg10Ps while it remains at the same position for Zn10Ag1Ps. The main characteristic peaks of Zn-O vibration at 584 cm^−1^ was observed for all three samples (Mahalakshmi et al., 2020). Additionally, there are some other peaks observed at 2166, 2042, 1573, 1383, 1203, 1075, and 640 cm^−1^ which may be originated from the different vibrational bands of phenolic, flavonoid, sugar, starch, citric acid, ascorbic acid, and plumbagin (Jyoti et al., 2016).

### 3.5 GC-MS/MS analysis

The major phytochemicals detected in PALE were dodecanoic acid, methyl ester, benzene, (3-octylundecyl)-, methyl stearate, methyl tetradecanoate, palmitic acid, methyl ester and others as evident from Table 3. These phytochemicals might have played a significant role in synthesis of the composites and their stabilization.

**TABLE 3:** Main compounds detected in PALE by GC-MS/MS.

Sr.No	Name of compound	Rt (min)	Molecular formula	Molecular mass (g/mol)	Retention index	Area (%)
1	Benzene, (3-octylundecyl)-	3.317	C_25_H_44_	344.62	2501	15.7
2	Disilicic acid (H6-Si2-O7), hexamethyl ester	4.252	C_12_H_30_O_7_Si_2_	342.53	1200	1.8
4	1-Nitro-β-d-arabinofuranose, tetraacetate	4.887	C_13_H_17_NO_11_	363.27	1301	0.4
5	Melezitose	5.124	C_18_H_32_O_16_	504.438	1801	1.3
6	trans-Isoeugenol	6.073	C_10_H_12_O	164.2011	1000	0.4
7	1,4-Benzenedicarboxylic acid, dimethyl ester	7.008	C_10_H_10_O	194.18	1001	6.5
8	Dodecanoic acid, methyl ester	7.066	C_13_H_26_O	214.3443	1301	27.3
9	Benzenemethanimine, α-phenyl-	8.092	C_13_H_11_N	181.2331	1300	2.0
10	Methyl tetradecanoate	8.532	C_15_H_30_O	242.3975	1501	6.5
11	2-Propenoic acid, 3-(4-hydroxyphenyl)-, methyl ester	8.892	C_10_H_10_O_3_	178.1846	1000	2.0
13	Nicotinamide, 2,6-dimethoxy-4-methyl-	9.743	C_9_H_12_N	196.203	900	0.7
14	1,4-benzenedicarboxylic acid, 2-hydroxyethyl methyl ester	9.846	C_11_H_12_O_5_	224.21	1101	0.6
15	(+)-Asarinin	10.074	C_20_H_18_O_6_	354.4	2001	0.5
16	Palmitic acid, methyl ester	10.566	C_17_H_34_O_2_	270.45	1700	8.3
17	2,3-Dimethyl-5-(trifluoromethyl)-1,4-benzenediol #	11.047	C_9_H_9_F_3_O_2_	206.16	901	0.6
18	16β-Hydroxyboldenone	11.258	C_19_H_26_O_2_	286.4	1900	0.6
19	Androsta-1,4-dien-3-one, 17-hydroxy-17-methyl-, (17α)-	11.544	C_20_H_28_O_2_	300.4	2001	0.5
20	(6-Isocodeine)/Morphinan, 7,8-didehydro-4,5-epoxy-3,6-bis [(trimethylsilyl)oxy]-, (5α,6α)-	11.687	C_18_H_21_NO_3_	299.4	1800	0.4
21	Methyl petroselinate	12.368	C_19_H_36_O_2_	296.5	1901	1.1
22	Oleic acid, methyl ester	12.424	C_19_H_36_O_2_	296.5	1900	0.6
23	8-Methylnonanoic acid, methyl ester	12.601	C_11_H_22_O	186.2912	1101	3.2
24	Methyl stearate	12.68	C_19_H_38_O_2_	298.5	1900	11.8
26	Nonanoic acid, 9-(o-propylphenyl)-, methyl ester	12.869	C_19_H_30_O_2_	290.45	1901	0.4
27	10,13-Octadecadiynoic acid, methyl ester	13.019	C_19_H_34_O_2_	294.5	1900	0.4
28	Carnegine (6,7-dimethoxy-1,2-dimethyl-3,4-dihydro-1H-isoquinoline)	13.195	C_13_H_19_NO_2_	221.29	1301	0.3
29	Benzene, 2-(1-decylundecyl)-1,4-dimethyl-	13.328	C_29_H_52_	400.7232	2900	0.3
30	Dihydroxanthin	14.066	C_17_H_24_O_5_	308.4	1701	0.3
31	n-Hexadecylsuccinic anhydride	14.466	C_20_H_36_O_3_	324.5	2002	0.3

### 3.6 Photocatalytic dye degradation

**FIGURE 7:**
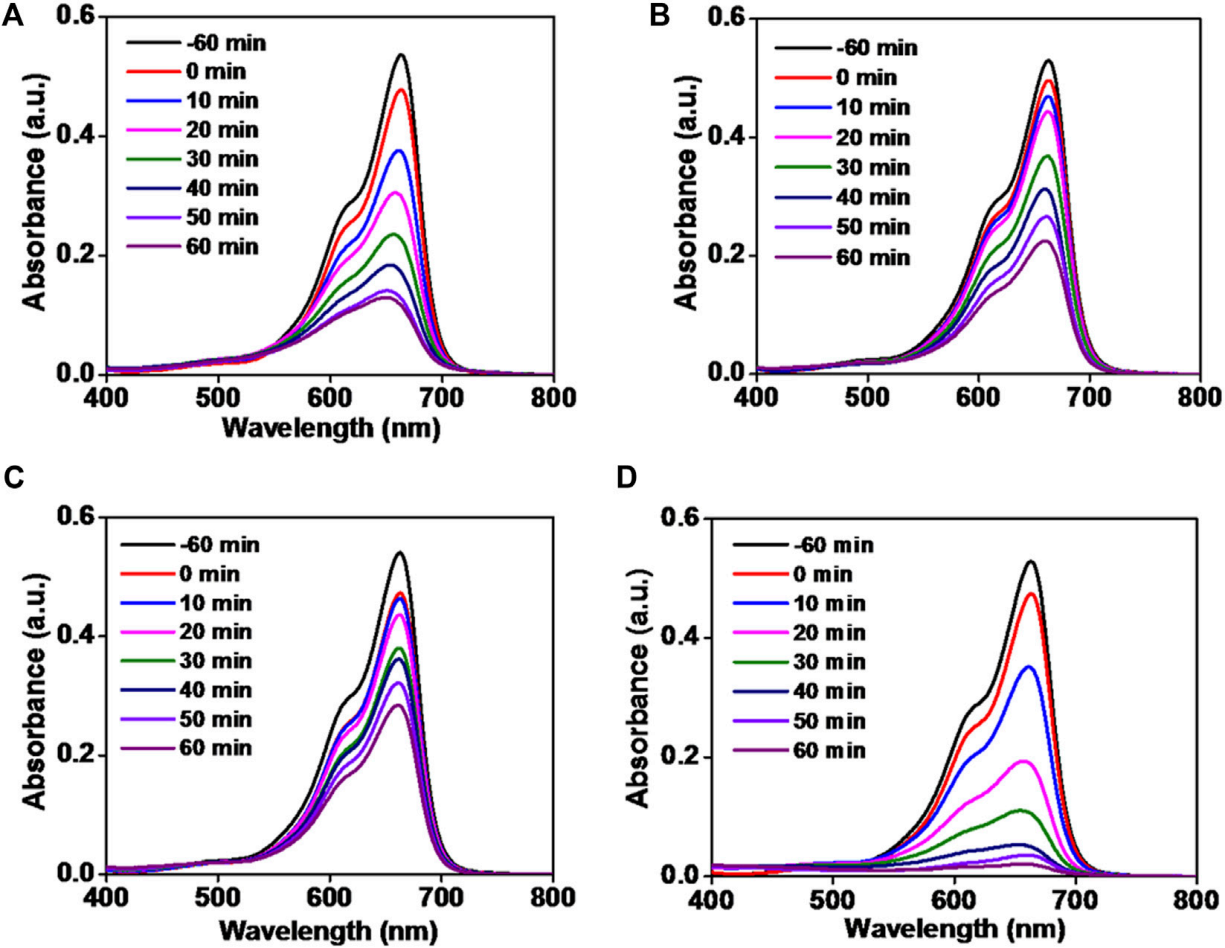
Absorption spectra of reduction of methylene blue dye by particles. **(A)** ZnOPs; **(B)** ZnOAg1Ps; **(C)** ZnOAg10Ps; **(D)** ZnO10Ag1Ps.

**FIGURE 8:**
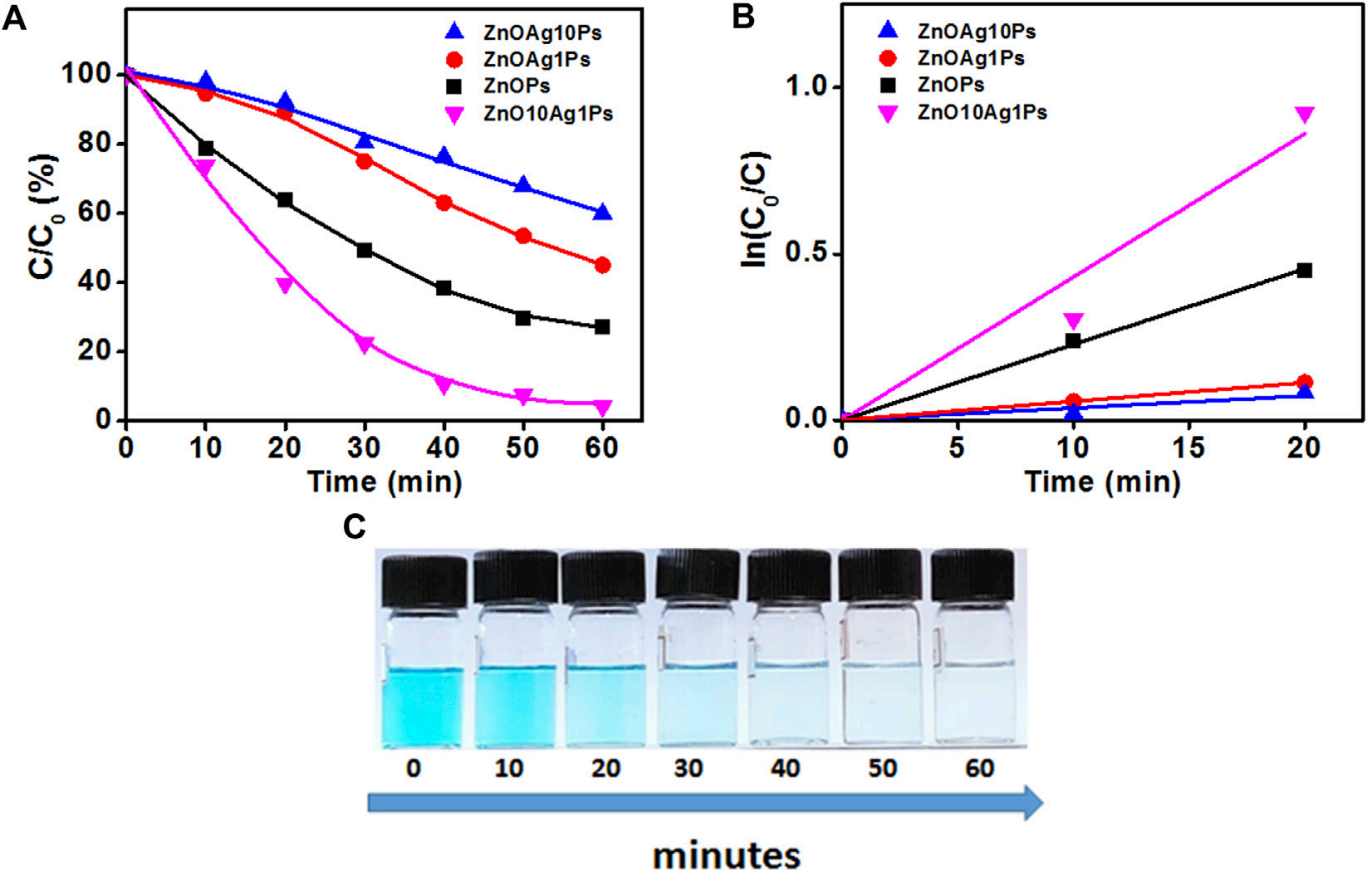
Degradation of MB in presence of particles. **(A)** Photocatalytic MB degradation by ZnOPs and ZnOAgPs under the UV light; **(B)** First-order kinetic constant for the degradation of MB under UV-light; **(C)**Visible colour change indicating MB degradation with time.

In order to investigate the photocatalytic degradation of MB dye, phytogenic ZnOPs and ZnOAgPs were mixed with MB dye solution and reacted for various time intervals. The photocatalytic activity of ZnOPs and ZnOAgPs is shown in Figure 7. The degradation of MB under UV irradiation in presence of ZnOPs and ZnOAgPs synthesized by PALE was monitored using an UV-visible spectrophotometer. The absorption maxima of MB was centered at 664 nm and shoulder peak at 614 nm in visible region (Figure 7). The main absorption peak steadily decreased and eventually approached the base line when treated with the particles as clearly evident from Figures 7A–D. The plot of ln (C_0_/C) *vs.* time for the catalytic degradation of MB is shown in Figure 8 while the visible colour change indicating dye degradation can be seen in Figure 8C. The experimental data fits well with the first order kinetic model with variable rate constants (k_1_) as given in Table 4. ZnOAg10Ps showed the maximum MB dye adsorption (12.5%), followed by ZnO10Ag1Ps and others. ZnO10Ag1Ps showed maximum MB dye degradation of 95.7% with a rate constant of 0.0463 s^−1^ followed by others.

**TABLE 4:** Percentage of adsorption of MB and first order kinetic constant of particles.

Nanoparticles	Adsorption (%)	Degradation (%)	k_1_ (s^−1^)
ZnOAg10Ps	12.5	40.4	0.0036
ZnOAg1Ps	6.5	55.1	0.0056
ZnO10Ag1Ps	10.3	95.7	0.0463
ZnOPs	10.99	72.7	0.0228

## 4 Discussion

Recently, ZnOPs have drawn much attention as promising photocatalyst which is mainly attributed to their attractive properties that include high UV light absorption, high exciton binding energy (60 meV), high electron mobility, tunable morphology, easy availability, economical production and nontoxic nature (Ansari et al., 2017; Kim and Jo, 2017). However, the chemical and physical methods of ZnOPs synthesis involve hazardous reaction conditions and toxic chemicals for reduction and stabilization (Adersh et al., 2015). Hence, PALE mediated biofabrication is a green route for synthesis of ZnOPs with photocatalytic properties (Kitture et al., 2015). However, the photocatalytic potential of ZnOPs is often limited due to fast recombination of photo-excited electron–hole pairs in the single-phase semiconductor and a low quantum yield. This further limits the application and commercialization potential of pristine ZnO for photocatalytic degradation process (Saravanam et al., 2015a; Lee et al., 2015). One of the prominent solutions to deal with this bottleneck is to dope noble metals in the ZnOPs for synergistic enhancement of the photocatalytic activity (Saravanam et al., 2015b; Ansari et al., 2013; Ansari et al., 2015). Hence, we have attempted to mix Ag in the biogenic ZnOPs at various concentrations that might offer several advantages. Ag was selected because of its attractive properties such as, high electrical and thermal conductivity, high work function, and cost effectiveness (Zeng et al., 2017; Gami et al., 2022). Although some of the studies give an account on the chemically synthesized core shell ZnO/Ag photocatalysts, there are no reports on Ag mixed ZnOPs from *P. auriculata* (Andrade et al., 2017; Gupta et al., 2017; Rafaie et al., 2017; Sampaio et al., 2017). The advantage of phytofabricated ZnOAgPs is the stable deposition of the Ag on the semiconductor’s active surface which is protected by the phytochemicals unlike the chemical methods where the leaching of Ag from the composite system can pollute the water apart from affecting the photocatalytic reactions due to reduction in active surface. Also, the biogenic stable ZnOAgPs might be resistant to photo-chemical corrosion that often results under adverse or extreme abiotic conditions (Li et al., 2013; Ma et al., 2017). These phytofabricated composites enhance the proximity of the interface between semiconductor and Ag which is important for transfer of charge and optimization of plasmon–exciton interactions (Das et al., 2015).

Kadam et al. (2018) reported that this strategy accelerates Fermi level equilibrium and can reduce the band gap energy that in turn can promote separation of the interfacial charge and accelerate electron transfer. Additionally, this can prevent interaction from harsh chemicals and hence restrict agglomeration and degradation of the metal particles (Ansari et al., 2014). Nanostructured noble metals serve as active reaction sites and efficient electron traps that oppose the charge carriers to recombine (Guy and Ozacar, 2016).

In our study, the UV-Vis peak of ZnOPs was prominent in the ZnOAgPs indicating the higher amount of ZnO in the composites. It is important to note that the absorption spectra of ZnOAgPs didn’t show superposition with the individual single-component particles which might be attributed to the strong electronic coupling between the Ag and ZnO (Satter et al., 2014). Ag mixed in the ZnO increased separation of charge, while the high ZnO content in the vicinity resulted in the increase in the refractive index of the surrounding medium which resulted in the shifting of the SPR band (Karmakar et al., 2020; Robkhob et al., 2020). Further, PL spectra provided strong scientific evidence of the distinct quenching of the emission in ZnOAgPs which was attributed to the efficient charge transfer from ZnO to Ag interface (Misra et al., 2014). Morphological analysis using HRTEM showed anisotropy among variously mixed structures. Spheres, rods, hexagons were observed. Our results are well in agreement with the ZnONPs synthesized using *Salvia officinalis* leaf extract (Abomuti et al., 2021). Ahmed et al. (2020) also synthesized similar Au/ZnO nanoparticles by using *Carya illinoinensis* leaf extract for enhanced photocatalytic degradation of Rhodamine B dye. In another study, ZnONPs synthesized by *Ziziphus jujuba* leaves extract also formed irregular agglomerated particles with variable shape and size where irregular flake-shape composites were formed with very small particles with an average particle size of 20 nm. Some particles were well-defined with spherical shape having an average particle size of 15 nm (Alharthi et al., 2021). The FTIR spectra confirmed the involvement of the active functional groups of the phytochemicals present in PALE for the synthesis and stabilization of the particles. Ameen et al. (2021) also reported similar involvement of the functional groups during synthesis of ZnONPs from *Acremonium potronii.*

Both ZnOPs and ZnOAgPs exhibited promising photocatalytic MB dye degradation which was found to be a function of reaction time. Phytogenic ZnONPs from aqueous extract of *Vitis rotundifolia* showed malachite green dye degradation after 150 min where the initial strong absorption peak visible at 620 nm gradually decreased with time (Brindhadevi et al., 2020). However, in case of ZnO10Ag1Ps the degradation of the MB was 95.7% within 60 min which was comparatively faster. Davar et al. (2015) reported photocatalytic degradation of methyl orange, methyl red and methylene blue dyes by the ZnONPs synthesized using lemon juice. On mixing with Ag the photocatalytic activity of ZnOPs against MB was increased which is in close agreement where AgNPs synthesized using Gongura leaves were decorated on to ZnONPs fabricated by hydrothermal route. The 5% AgNPs containing ZnO composites photodegraded (MB) dye up to 99.21% within 75 min under UV light irradiation (Jadav et al., 2020). The concentration of MB decreased with the increase in time which was due to redox reaction. The superior photocatalytic activity in mixed ZnOPs might be attributed to the creation of additional energy levels positioned between the VB, and CB of the ZnO, that resulted in the dopant incorporation mediated extension of the light absorption range (Chaudhari et al., 2022). Moreover, creating more defect levels within ZnO helps to produce efficient traps of e‾, which in turn decreases the possibility of the recombination rate resulting in the rapid formation of active radicals (Karthik et al., 2022). Previous reports establish the mechanism for the same which indicate light absorption mediated VB to CB transfer of the photoexcited electrons upon irradiation. Therefore, the transit of the photoexcited VB electrons to the CB/impurity energy levels from the host lattice results in the generation of high amount of O_2_—associated with *d-d* transitions. During this time, transfer of the VB photoexcited holes to the surface occurs that interrelate with water molecules, eventually generating large amount of •OH oxidative radical species. During the oxidation, the organic pollutants can catch the photoexcited holes. Hence, being tough and non selective, the generated reactive oxygen species can result in promising dye degradation at the surface of the photocatalyst. Similar results were noted with ZnO based nanocomposites synthesized using *S. lycopersicum,* coconut husk, *Allium cepa* L, *Syzygium cumini*, *Ruellia tuberose, Artocarpus heterophyllus* for degradation of chemical dyes such as, Congo red, methylene blue, metanil yellow, crystal violet, and malachite green (Vidya et al., 2017; Rajkumar et al., 2019; Preethi et al., 2020; Sadiq et al., 2021; Vasantharaj et al., 2021; Priyadharshini et al., 2022). In view of the background, it is evident that Ag mixed phytogenic ZnOPs mediated dye removal can be a powerful strategy for dye contaminated wastewater treatment.

## 5 Conclusion

The current study reports the facile synthesis of pristine and Ag-mixed ZnOPs employing an environmentally benign, rapid and efficient route using *P. auriculata* leaf extract. The concentrations of the precursor metal salt concentrations were varied to get three different composites denoted as ZnOAg1Ps, ZnOAg10Ps, and ZnO10Ag1Ps. The particles were polydispersed with spherical, rod, hexagonal and irregular shapes. The phytochemical analysis revealed significant amount of polyphenols, flavonoids, starch, reducing sugar, citric acid and plumbagin in PALE that might play a critical role in the synthesis and the stabilization of the nanoparticles. The structural and optical analysis confirmed the formation of crystalline pure ZnOPs as well as Ag doping. Both phytogenic ZnOPs and ZnOAgPs showed superior photocatalytic MB dye degrading ability. Maximum MB dye degradation up to 95.7% with a rate constant of 0.0463 s^−1^ was exhibited by ZnO10Ag1Ps. Hence, phytofabricated Ag mixed ZnOPs can help to develop promising strategy for treating hazardous dye contaminated industrial effluents to ensure clean environment.

## Data Availability

The original contributions presented in the study are included in the article/supplementary material, further inquiries can be directed to the corresponding authors.

## References

[B1] Abomuti M. A. Danish E. Y. Firoz A. Hasan N. Malik M. A. (2021). Green synthesis of zinc oxide nanoparticles using *Salvia officinalis* leaf extract and their photocatalytic and antifungal activities. Biology 10, 1075. 10.3390/biology10111075 34827068 PMC8614830

[B2] Adersh A. Kulkarni A. R. Ghosh S. More P. Chopade B. A. Gandhi M. N. (2015). Surface defect rich ZnO quantum dots as antioxidant inhibiting α-amylase and α-glucosidase: A potential anti-diabetic nanomedicine. J. Mat. Chem. B 3, 4597–4606. 10.1039/C5TB00407A 32262403

[B3] Ahmad M. Rehman W. Khan M. M. Qureshi M. T. Gul A. Haq S. (2020). Phytogenic fabrication of ZnO and gold decorated ZnO nanoparticles for photocatalytic degradation of rhodamine B. J. Environ. Chem. Eng. 9, 104725. 10.1016/j.jece.2020.104725

[B4] Alharthi M. N. Ismail I. Bellucci S. Salam M. A. (2021). Green synthesis of zinc oxide nanoparticles by *Ziziphus jujuba* leaves extract: Environmental application, kinetic and thermodynamic studies. J. Phys. Chem. Solids 158, 110237. 10.1016/j.jpcs.2021.110237

[B5] Ameen F. Dowoud T. Alnadhari S. (2021). Ecofriendly and low cost synthesis of ZnO nanoparticles from *Acremonium potronii* for the photocatalytic degradation of azo dyes. Environ. Res. 202, 111700. 10.1016/j.envres.2021.111700 34274331

[B6] Andrade G. R. S. Nascimento C. C. Lima Z. M. Neto E. T. Costa L. P. Gimenez I. F. (2017). Star-shaped ZnO/Ag hybrid nanostructures for enhanced photocatalysis and antibacterial activity. Appl. Surf. Sci. 399, 573–582. 10.1016/j.apsusc.2016.11.202

[B7] Ansari S. A. Ansari S. G. Foaud H. Cho M. H. (2017). Facile and sustainable synthesis of carbon-doped ZnO nanostructures towards the superior visible light photocatalytic performance. New J. Chem. 41, 9314–9320. 10.1039/C6NJ04070E

[B8] Ansari S. A. Khan M. M. Ansari M. O. Lee J. Cho M. H. (2013). Biogenic synthesis, photocatalytic, and photoelectrochemical performance of Ag–ZnO nanocomposite. J. Phys. Chem. C 117, 27023–27030. 10.1021/jp410063p

[B9] Ansari S. A. Khan M. M. Ansari M. O. Lee J. Cho M. H. (2015). Gold nanoparticles-sensitized wide and narrow band gap TiO_2_ for visible light applications: A comparative study. New J. Chem. 39, 4708–4715. 10.1039/C5NJ00556F

[B10] Ansari S. A. Khan M. M. Lee J. Cho M. H. (2014). Highly visible light active Ag@ZnO nanocomposites synthesized by gel-combustion route. J. Ind. Eng. Chem. 20, 1602–1607. 10.1016/j.jiec.2013.08.006

[B11] Basri H. H. Talib R. A. Sukor R. Othman S. H. Ariffin H. (2020). Effect of synthesis temperature on the size of ZnO nanoparticles derived from pineapple peel extract and antibacterial activity of ZnO–starch nanocomposite films. Nanomaterials 10, 1061. 10.3390/nano10061061 PMC7352361 32486281

[B12] Bloch K. Webster T. J. Ghosh S. (2020). “Mycogenic synthesis of metallic nanostructures and their use in dye degradation,” in Photocatalytic degradation of dyes: Current trends and future. Editors Dave S. Das J. Shah M. (Amsterdam, Netherlands: Elsevier), 509–525. 10.1016/B978-0-12-823876-9.00014-7

[B13] Brindhadevi K. Samuel M. S. Verma T. N. Vasantharaj S. Sathiyavimal S. Saravanan M. (2020). Zinc oxide nanoparticles (ZnONPs) -induced antioxidants and photocatalytic degradation activity from hybrid grape pulp extract (HGPE). Biocatal. Agric. Biotechnol. 124, 101730. 10.1016/j.bcab.2020.101730

[B14] Brus L. (1986). Electronic wave functions in semiconductor clusters: Experiment and theory. J. Phys. Chem. 90, 2555–2560. 10.1021/j100403a003

[B15] Chaudhari A. Kaida T. Desai H. B. Ghosh S. Bhatt R. P. Tanna A. R. (2022). Dye degradation and antimicrobial applications of manganese ferrite nanoparticles synthesized by plant extracts. Chem. Phys. Impact 5, 100098. 10.1016/j.chphi.2022.100098

[B16] Daneshvar N. Ayazloo M. Khataee A. R. Pourhassan M. (2007). Biological decolorization of dye solution containing malachite green by microalgae *Cosmarium* sp. Bioresour. Technol. 98, 1176–1182. 10.1016/j.biortech.2006.05.025 16844368

[B17] Das S. Sinha S. Suar M. Yun S. Mishra A. Tripathy S. K. (2015). Solar-photocatalytic disinfection of *Vibrio cholerae* by using Ag@ZnO core–shell structure nanocomposites. J. Photochem. Photobiol. B Biol. 142, 68–76. 10.1016/j.jphotobiol.2014.10.021 25523714

[B18] Davar F. Majedi A. Mirzaei A. (2015). Green synthesis of ZnO nanoparticles and its application in the degradation of some dyes. J. Am. Ceram. Soc. 98, 1739–1746. 10.1111/jace.13467

[B19] Din M. I. Khalid R. Najeeb J. Hussain Z. (2021). Fundamentals and photocatalysis of methylene blue dye using various nanocatalytic assemblies- a critical review. J. Clean. Prod. 298, 126567. 10.1016/j.jclepro.2021.126567

[B20] Fagier M. A. (2021). Plant-mediated biosynthesis and photocatalysis activities of zinc oxide nanoparticles: A prospect towards dyes mineralization. J. Nanotechnol. 2021, 1–15. 10.1155/2021/6629180

[B21] Gami B. Bloch K. Mohammed S. M. Karmakar S. Shukla S. Asok A. (2022). *Leucophyllum frutescens* mediated synthesis of silver and gold nanoparticles for catalytic dye degradation. Front. Chem. 10, 932416. 10.3389/fchem.2022.932416 36247678 PMC9557002

[B22] Gautam S. Kaithwas G. Bharagava R. N. Saxena G. (2017). “Pollutants in tannery wastewater, their pharmacological effects and bioremediation approaches for human health protection and environmental safety,” in Environmental pollutants and their bioremediation approaches. Editor Bharagava R. N. (Boca Raton, FL, USA: CRC Press, Taylor & Francis Group), 369–396. 10.1201/9781315173351-14

[B23] Ghosh S. Gurav S. P. Harke A. N. Chacko M. J. Joshi K. A. Dhepe A. (2016a). *Dioscorea oppositifolia* mediated synthesis of gold and silver nanoparticles with catalytic activity. J. Nanomed. Nanotechnol. 7, 1000398. 10.4172/2157-7439.1000398

[B24] Ghosh S. Sarkar B. Kaushik A. Mostafavi E. (2022). Nanobiotechnological prospects of probiotic microflora: Synthesis, mechanism and applications. Sci. Total Environ. 838, 156212. 10.1016/j.scitotenv.2022.156212 35623529

[B25] Govindan L. Anbazhagan S. Altemimi B. A. Lakshminarayanan K. Kuppan S. Pratap-Singh A. (2020). Efficacy of antimicrobial and larvicidal activities of green synthesized silver nanoparticles using leaf extract of *Plumbago auriculata* Lam. Plants 9, 1577. 10.3390/plants9111577 PMC7698226 33202641

[B26] Gupta J. Mohapatra J. Bahadur D. (2017). Visible light driven mesoporous Ag-embedded ZnO nanocomposites: Reactive oxygen species enhanced photocatalysis, bacterial inhibition and photodynamic therapy. Dalton Trans. 46, 685–696. 10.1039/C6DT03713E 27896346

[B27] Guy N. Ozacar M. (2016). The influence of noble metals on photocatalytic activity of ZnO for Congo red degradation. Int. J. Hydrogen Energy 41, 20100–20112. 10.1016/j.ijhydene.2016.07.063

[B28] Hashmi S. S. Shah M. Muhammad W. Ahmad A. Ullah M. A. Nadeem M. (2021). Potentials of phyto-fabricated nanoparticles as ecofriendly agents for photocatalytic degradation of toxic dyes and waste water treatment, risk assessment and probable mechanism. J. Indian Chem. Soc. 98, 100019. 10.1016/j.jics.2021.100019

[B29] Israni S. A. Kapadia N. S. Lahiri S. K. Yadav G. Shah M. B. (2010). An UV-Vis spectrophotometric method for the estimation of plumbagin. Int. J. ChemTech. Res. 2, 856–859.

[B30] Jadav P. Shinde S. Suryawanshi S. S. Teli S. B. Patil P. S. Ramteke A. A. (2020). Green AgNPs decorated ZnO nanocomposites for dye degradation and antimicrobial applications. Eng. Sci. 12, 79–94. 10.30919/es8d1138

[B31] Jain N. Bharagava A. Majumdar S. Tarafdar J. C. Panwar J. (2011). Extracellular biosynthesis and characterization of silver nanoparticles using *Aspergillus flavus* NJP08: A mechanism perspective. Nanoscale 3, 635–641. 10.1039/c0nr00656d 21088776

[B32] Jamdade D. A. Rajpali D. Joshi K. A. Kitture R. Kulkarni A. S. Shinde V. S. (2019). *Gnidia glauca*- and *Plumbago zeylanica*-mediated synthesis of novel copper nanoparticles as promising antidiabetic agents. Adv. Pharmacol. Sci. 2019, 1–11. 10.1155/2019/9080279 PMC6388358 30886631

[B33] Jangir L. K. Kumari Y. Kumar A. Kumar M. Awasthi K. (2017). Investigation of luminescence and structural properties of ZnO nanoparticles, synthesized with different precursors. Mat. Chem. Front. 7, 1413–1421. 10.1039/C7QM00058H

[B34] Jyoti K. Baunthiyal M. Singh A. (2016). Characterization of silver nanoparticles synthesized using *Urtica dioica* Linn. leaves and their synergistic effects with antibiotics. J. Radiat. Res. Appl. Sci. 9, 217–227. 10.1016/j.jrras.2015.10.002

[B35] Kadam A. N. Bhopate D. P. Kondalkar V. V. Majhi S. M. Bathula C. D. Tran A. V. (2018). Facile synthesis of Ag-ZnO core-shell nanostructures with enhanced photocatalytic activity. J. Ind. Eng. Chem. 61, 78–86. 10.1016/j.jiec.2017.12.003

[B36] Kannan P. John S. A. (2008). Synthesis of mercaptothiadiazole-functionalized gold nanoparticles and their self-assembly on Au substrates. Nanotechnology 19, 085602. 10.1088/0957-4484/19/8/085602 21730726

[B37] Karmakar S. Ghosh S. Kumbhakar P. (2020). Enhanced sunlight driven photocatalytic and antibacterial activity of flower-like ZnO@MoS_2_ nanocomposite. J. Nanopart. Res. 22, 11. 10.1007/s11051-019-4710-3

[B38] Karthik K. V. Raghu A. V. Reddy K. R. Ravishankar R. Sangeeta M. Shetti N. P. (2022). Green synthesis of Cu-doped ZnO nanoparticles and its application for the photocatalytic degradation of hazardous organic pollutants. Chemosphere 287, 132081. 10.1016/j.chemosphere.2021.132081 34500333

[B39] Khan M. E. (2021). State-of-the-art developments in carbon-based metal nanocomposites as a catalyst: Photocatalysis. Nanoscale Adv. 3 (7), 1887–1900. 10.1039/d1na00041a 36133084 PMC9418201

[B40] Khan M. M. Saadah N. H. Khan M. E. Harunsani M. H. Tan A. L. Cho M. H. (2019a). Phytogenic synthesis of band gap-narrowed ZnO nanoparticles using the bulb extract of *Costus woodsonii* . Bionanoscience 9, 334–344. 10.1007/s12668-019-00616-0

[B41] Khan M. M. Saadah N. H. Khan M. E. Harunsani M. H. Tan A. L. Cho M. H. (2019b). Potentials of *Costus woodsonii* leaf extract in producing narrow band gap ZnO nanoparticles. Mat. Sci. Semicond. Process 91, 194–200. 10.1016/j.mssp.2018.11.030

[B42] Kim M. Jo W. K. (2017). Purification of aromatic hydrocarbons using Ag–multiwall carbon nanotube–ZnO nanocomposites with high performance. J. Ind. Eng. Chem. 47, 94–101. 10.1016/j.jiec.2016.11.018

[B43] Kitture R. Chordiya K. Gaware S. Ghosh S. More P. A. Kulkarni P. (2015). ZnO nanoparticles-red sandalwood conjugate: A promising anti-diabetic agent. J. Nanosci. Nanotechnol. 15, 4046–4051. 10.1166/jnn.2015.10323 26369011

[B44] Kumar R. Rashid J. Barakat M. A. (2015). Zero valent Ag deposited TiO_2_ for the efficient photocatalysis of methylene blue under UV-C light irradiation. Colloids Interface Sci. Commun. 5, 1–4. 10.1016/j.colcom.2015.05.001

[B45] Lee H. J. Kim J. H. Park S. S. Hong S. S. Lee G. D. (2015). Degradation kinetics for photocatalytic reaction of methyl orange over Al-doped ZnO nanoparticles. J. Ind. Eng. Chem. 25, 199–206. 10.1016/j.jiec.2014.10.035

[B46] Li J. Cushing S. K. Bright J. Meng F. Senty T. R. Zheng P. (2013). Ag@Cu_2_O core-shell nanoparticles as visible-light plasmonic photocatalysts. ACS Catal. 3, 47–51. 10.1021/cs300672f

[B47] Li S. Shen Y. Xie A. Yu X. Qiu L. Zhnag L. (2007). Green synthesis of silver nanoparticles using *Capsicum annuum* L. extract. Green Chem. 9, 852–858. 10.1039/B615357G

[B48] Luximon-Ramma A. Bahorun T. Soobrattee M. A. Aruoma O. I. (2002). Antioxidant activities of phenolic, proanthocyanidin, and flavonoid components in extracts of *Cassia fistula* . J. Agric. Food Chem. 50, 5042–5047. 10.1021/jf0201172 12188605

[B49] Lv J. Gong W. Huang K. Zhu J. Meng F. Song X. (2011). Effect of annealing temperature on photocatalytic activity of ZnO thin films prepared by sol–gel method. Superlattices Microstruct. 50, 98–106. 10.1016/j.spmi.2011.05.003

[B50] Ma X. Li H. Liu T. Du S. Qiang Q. Wang Y. (2017). Comparison of photocatalytic reaction-induced selective corrosion with photocorrosion: Impact on morphology and stability of Ag-ZnO. Appl. Catal. B Environ. 201, 348–358. 10.1016/j.apcatb.2016.08.029

[B51] Mahalakshmi S. Hema N. Vijaya P. P. (2020). *In vitro* biocompatibility and antimicrobial activities of zinc oxide nanoparticles (ZnONPs) prepared by chemical and green synthetic route- A comparative study. Bionanoscience 10, 112–121. 10.1007/s12668-019-00698-w

[B52] Mandeep and Shukla P. (2020). Microbial nanotechnology for bioremediation of industrial wastewater. Front. Microbiol. 11, 590631. 10.3389/fmicb.2020.590631 33224126 PMC7667373

[B53] Mani S. Bharagava R. N. (2017). Isolation, screening and biochemical characterization of bacteria capable of crystal violet dye decolorization. Int. J. Adv. Sci. Res. 2, 70–75.

[B54] Miller G. L. (1959). Use of dinitrosalicylic acid reagent for determination of reducing sugar. Anal. Chem. 31, 426–428. 10.1021/ac60147a030

[B55] Misra M. Kapur P. Nayak M. Singla M. (2014). Synthesis and visible photocatalytic activities of a Au@Ag@ZnO triple layer core–shell nanostructure. New J. Chem. 38, 4197–4203. 10.1039/C4NJ00569D

[B56] Mohammad A. Karim M. R. Khan M. E. AlSukaibi A. K. D. Yoon T. (2022). Eco-benign fabrication of silver nanoparticle-modified zeolitic imidazolate framework and construction of a non-enzymatic electrochemical sensor. Mater. Today Sustain. 19, 100182. 10.1016/j.mtsust.2022.100182

[B57] Naseer M. Aslam U. Khalid B. Chen B. (2020). Green route to synthesize zinc oxide nanoparticles using leaf extracts of *Cassia fistula* and *Melia azadarach* and their antibacterial potential. Sci. Rep. 10, 9055. 10.1038/s41598-020-65949-3 32493935 PMC7270115

[B58] Nitnavare R. Bhattacharya J. Thongmee S. Ghosh S. (2022). Photosynthetic microbes in nanobiotechnology: Applications and perspectives. Sci. Total Environ. 841, 156457. 10.1016/j.scitotenv.2022.156457 35662597

[B59] Preethi S. Abrana K. Nithyasri M. Kishore P. Deepika K. Ranjithkumar R. (2020). Synthesis and characterization of chitosan/zinc oxide nanocomposite for antibacterial activity onto cotton fabrics and dye degradation applications. Int. J. Biol. Macromol. 164, 2779–2787. 10.1016/j.ijbiomac.2020.08.047 32777425

[B60] Priyadharshini S. S. Shubha J. P. Shivalingappa J. Adil S. F. Kuniyil M. Hatshan M. R. (2022). Photocatalytic degradation of methylene blue and metanil yellow dyes using green synthesized zinc oxide (ZnO) nanocrystals. Crystals 12, 22–16. 10.3390/cryst12010022

[B61] Rafaie H. A. Nor R. M. Azmina M. S. Ramli N. I. T. Mokamed R. (2017). Decoration of ZnO microstructures with Ag nanoparticles enhanced the catalytic photodegradation of methylene blue dye. J. Environ. Chem. Eng. 5, 3963–3972. 10.1016/j.jece.2017.07.070

[B62] Rajkumar K. S. Arun S. Babu M. D. Balaji P. Sivasubramanian S. Vignesh V. (2019). Facile biofabrication, characterization, evaluation of photocatalytic, antipathogenic activity and *in vitro* cytotoxicity of zinc oxide nanoparticles. Biocatal. Agric. Biotechnol. 22, 101436. 10.1016/j.bcab.2019.101436

[B63] Robkhob P. Ghosh S. Bellare J. Jamdade D. Tang I. M. Thongmee S. (2020). Effect of silver doping on antidiabetic and antioxidant potential of ZnO nanorods. J. Trace Elem. Med. Biol. 58, 126448. 10.1016/j.jtemb.2019.126448 31901726

[B64] Sadasivam S. Manickam A. (2008). Biochemical methods. 3rd ed. New Delhi, India: New Age International.

[B65] Sadiq H. Sher F. Sehar S. Lima E. C. Zhuang S. Iqbal H. M. N. (2021). Green synthesis of ZnO nanoparticles from *Syzygium cumini* leaves extract with robust photocatalysis applications. J. Mol. Liq. 335, 116567. 10.1016/j.molliq.2021.116567

[B66] Saffran M. Denstedt O. F. (1948). A rapid method for the determination of citric acid. J. Biol. Chem. 175, 849–855. 10.1016/s0021-9258(18)57202-1 18880780

[B67] Saleh R. Djaja N. F. (2014). UV light photocatalytic degradation of organic dyes with Fe-doped ZnO nanoparticles. Superlattices Microstruct. 74, 217–233. 10.1016/j.spmi.2014.06.013

[B68] Salunke G. R. Ghosh S. Kumar R. J. S. Khade S. Vashisth P. Kale T. (2014). Rapid efficient synthesis and characterization of silver, gold and bimetallic nanoparticles from the medicinal plant *Plumbago zeylanica* and their application in biofilm control. Int. J. Nanomedicine 4, 2635–2653. 10.2147/IJN.S59834 PMC4043712 24920901

[B69] Sampaio M. J. Lima M. J. Baptistata D. L. Silva A. M. T. Silva C. G. Faria J. L. (2017). Ag-loaded ZnO materials for photocatalytic water treatment. Chem. Eng. J. 318, 95–102. 10.1016/j.cej.2016.05.105

[B70] Saoud K. Alsoubaihi R. Bensalah N. Bora T. Bertino M. Dutta J. (2015). Synthesis of supported silver nano-spheres on zinc oxide nanorods for visible light photocatalytic applications. Mat. Res. Bull. 63, 134–140. 10.1016/j.materresbull.2014.12.001

[B71] Saraf R. Shivakumara C. Behera S. Nagabhushana H. Dhananjaya N. (2015). Facile synthesis of PbWO_4_: Applications in photoluminescence and photocatalytic degradation of organic dyes under visible light. Spectrochimica Acta Part A Mol. Biomol. Spectrosc. 136, 348–355. 10.1016/j.saa.2014.09.038 25448939

[B72] Saravanam R. Khan M. M. Gupta V. K. Mosquera E. Gracia F. Narayanan V. (2015a). ZnO/Ag/CdO nanocomposite for visible light-induced photocatalytic degradation of industrial textile effluents. J. Colloid Interface Sci. 452, 126–133. 10.1016/j.jcis.2015.04.035 25935283

[B73] Saravanam R. Khan M. M. Gupta V. K. Mosquera E. Gracia F. Narayanan V. (2015b). ZnO/Ag/Mn_2_O_3_ nanocomposite for visible light-induced industrial textile effluent degradation, uric acid and ascorbic acid sensing and antimicrobial activity. RSC Adv. 5, 34645–34651. 10.1039/c5ra02557e

[B74] Satter S. S. Hoque M. Rahman M. M. Yousuf M. Mollah A. Susan M. (2014). An approach towards the synthesis and characterization of ZnO@Agcore@shell nanoparticles in water-in-oil microemulsion. RSC Adv. 4, 20612–20615. 10.1039/C4RA01046A

[B75] Sharwani A. A. Narayanan K, B. Khan M. E. Han S. S. (2022). Photocatalytic degradation activity of goji berry extract synthesized silver loaded mesoporous zinc oxide (Ag@ZnO) nanocomposites under simulated solar light irradiation. Sci. Rep. 12, 10017. 10.1038/s41598-022-14117-w 35705651 PMC9200859

[B76] Sharwani A. A. Narayanan K, B. Khan M. E. Han S. S. (2021). Sustainable fabrication of silver-titania nanocomposites using goji berry (*Lycium barbarum* L.) fruit extract and their photocatalytic and antibacterial applications. Arab. J. Chem. 14 (12), 103456. 10.1016/j.arabjc.2021.103456

[B77] Shende S. Joshi K. A. Kulkarni A. S. Charolkar C. Shinde V. S. Parihar V. S. (2018). *Platanus orientalis* leaf mediated rapid synthesis of catalytic gold and silver nanoparticles. J. Nanomed. Nanotechnol. 9, 1000494. 10.4172/2157-7439.1000494

[B78] Singh K. Naidoo Y. Baijnath H. (2018). A comprehensive review on the genus *Plumbago* with focus on *Plumbago auriculata (Plumbaginaceae*). Afr. J. Tradit. Complement. Altern. Med. 15, 199–215. 10.21010/ajtcam.v15i1.21

[B79] Thakur D. Sharma A. Rana D. S. Thakur N. Singh D. Tamulevicius T. (2020). Facile synthesis of silver-doped zinc oxide nanostructures as efficient scaffolds for detection of p-nitrophenol. Chemosensors 8 (4), 108. 10.3390/chemosensors8040108

[B80] Thayumanavan B. Sadasivam S. (1984). Physicohemical basis for the preferential uses of certain rice varieties. Plant Food. Hum. Nutr. 34, 253–259. 10.1007/BF01126554

[B81] Tripathy A. Raichur A. M. Chandrasekaran N. Prathna T. C. Mukherjee A. (2010). Process variables in biomimetic synthesis of silver nanoparticles by aqueous extract of *Azadirachta indica* (Neem) leaves. J. Nanopart. Res. 12, 237–246. 10.1007/s11051-009-9602-5

[B82] Vasantharaj S. Sathiyavimal S. Senthilkumar P. Kalpana V. N. Rajalakshmi G. Alsehli M. (2021). Enhanced photocatalytic degradation of water pollutants using bio-green synthesis of zinc oxide nanoparticles (ZnONPs). J. Environ. Chem. Eng. 9, 105772. 10.1016/j.jece.2021.105772

[B83] Vidya C. Manjunatha C. Chandraprabha M. N. Rajshekar M. Antony R. M. A. L. (2017). Hazard free green synthesis of ZnO nano-photocatalyst using *Artocarpus heterophyllus* leaf extract for the degradation of Congo red dye in water treatment applications. J. Environ. Chem. Eng. 5, 3172–3180. 10.1016/j.jece.2017.05.058

[B84] Wolfe K. Wu X. Liu R. H. (2003). Antioxidant activity of apple peels. J. Agric. Food Chem. 51, 609–614. 10.1021/jf020782a 12537430

[B85] Zachariah A. Baiju K. V. Shukla S. Deepa K. S. James J. Warrier K. G. K. (2008). Synergistic effect in photocatalysis as observed for mixed-phase nanocrystalline titania processed via sol-gel solvent mixing and calcination. J. Phys. Chem. C 112, 11345–11356. 10.1021/jp712174y

[B86] Zeng C. Yuan L. Li X. Goa C. Wang H. (2017). Fabrication of urchin-like Ag/ZnO hierarchical nano/microstructures based on galvanic replacement mechanism and their enhanced photocatalytic properties. Surf. Interface Anal. 49, 599–606. 10.1002/sia.6198

